# Gene model for the ortholog of *ABCB7* in *Drosophila funebris*

**DOI:** 10.17912/micropub.biology.002346

**Published:** 2026-09-24

**Authors:** Summer Dieterle, Pablo Chialvo, Clare Scott Chialvo

**Affiliations:** 1 Biology, Appalachian State University, Boone, North Carolina USA

## Abstract

We developed a gene model for the ortholog of the *Drosophila melanogaster* *ATP binding cassette subfamily B member 7* ( *ABCB7* ) gene in the ASM1890182v1 Genome Assembly (GenBank Accession: GCA_018901825.1 ) of *Drosophila funebris* . This ortholog was characterized as part of a developing dataset for a comparative study of detoxification gene family evolution in the *immigrans* - *tripunctata* radiation of the genus *Drosophila* using an adapted Genomics Education Partnership gene annotation protocol for Course-based Undergraduate Research Experiences.

**Figure 1. Genomic neighborhood and gene model for ABCB7 ortholog in D. funebris:** (A) **Synteny comparison of the genomic neighborhoods for *ABCB7* in *Drosophila melanogaster* and *Drosophila funebris* .** Thin underlying arrows indicate which DNA strand the target gene, *ABCB7* , is located on in *D. melanogaster* (top) and *D. funebris* (bottom). The thin arrows pointing to the right indicate that *ABCB7* is on the positive strand in both *D. melanogaster* and *D. funebris.* The wide gene arrows pointing in the same direction as *ABCB7* are on the same strand relative to the thin underlying arrows, while wide gene arrows pointing in the opposite direction of *ABCB7* are on the opposite strand relative to the thin underlying arrows. White gene arrows in *D. funebris* indicate orthology to the corresponding gene in *D. melanogaster* . Other colors of arrows indicate: black = non-orthology and grey = present in both neighborhoods but not syntenic. Gene symbols given in the *D. funebris* gene arrows indicate the orthologous gene in *D. melanogaster* , while the locus identifiers are specific to *D. funebris* . (B) **Gene Model in GEP UCSC Track Data Hub** (Raney et al., 2014). The coding-regions of *ABCB7* in *D. funebris* are displayed in the User Supplied Track (red); coding sequences (CDS) are depicted by thick rectangles and introns by thin lines with arrows indicating the direction of transcription. Subsequent evidence tracks include Spaln of *D. melanogaster* Proteins (purple, alignment of Ref-Seq proteins from *D. melanogaster* ), Coding Regions Predicted by Augustus (dark blue), GeMoMa (teal), and NSCAN PASA-EST (dark green), mapping of *D. melanogaster* CDS sequences using *tblastn* (grey to black), and RNA-Seq from mixed sex adult flies (brown; alignment of Illumina RNA-Seq reads from *D. funebris* – Erlenbach et al., 2023). (C) **Dot Plot of ABCB7-PA in *D. melanogaster* ( *x* -axis) vs. the orthologous peptide in *D. funebris* ( *y* -axis).** Amino acid number is indicated along the left and bottom; CDS number is indicated along the top and right, and CDSs are also highlighted with alternating colors. Line breaks in the dot plot indicate areas of with low sequence identity between species.

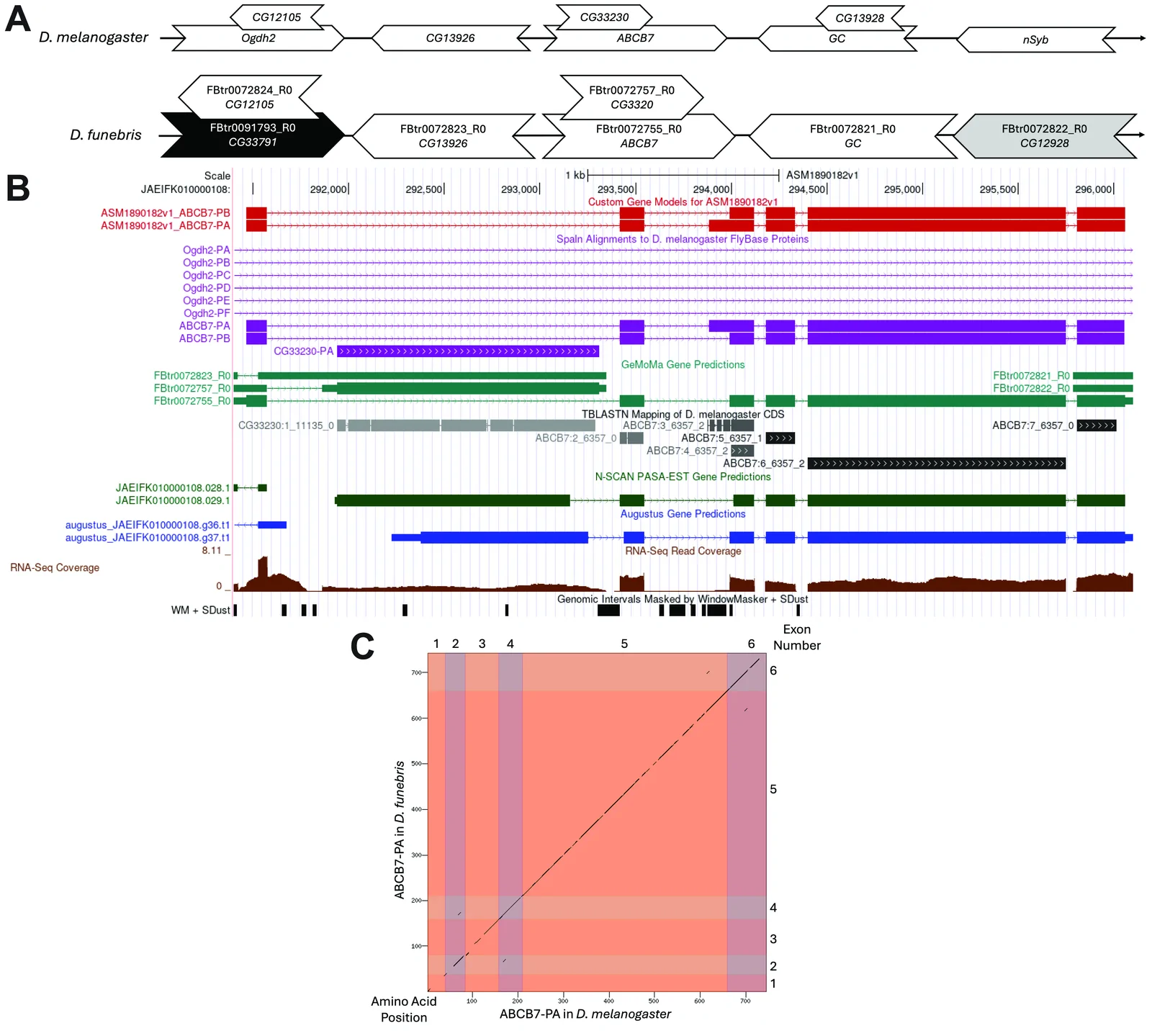



## Description

***Introduction***

**Table:** 

* This article reports a predicted gene model generated by undergraduate work using a structured gene model annotation protocol defined by the Genomics Education Partnership (GEP; thegep.org ) for Course-based Undergraduate Research Experience (CURE). The following information in quotes may be repeated in other articles submitted by participants using the same GEP CURE protocol for annotating Drosophila species orthologs of Drosophila melanogaster detoxification genes. * ** ** “Within insects, detoxifying xenobiotics and host secondary metabolites is a three-phase process that involves functionalization, conjugation, and excretion of these compounds. Expansions of known detoxification gene families ( *e.g.* , cytochrome P450s) are associated with diet breadth and insecticide resistance (Ranson et al., 2002; Després et al., 2007; Rane et al., 2016). With the increasing availability of high-quality genomes for non-model organisms, including *Drosophila* species beyond *D. melanogaster* , it is now possible to perform large scale comparative studies (Robinson et al., 2011; Kim et al., 2021; Threfall and Baxter, 2021). Careful manual annotation and curation of gene models can improve upon computational gene predictions in non-model species, which aids the accuracy of studies on gene and genome evolution (Mudge and Harrow, 2016; Tello-Ruiz et al., 2019). To aid in these annotations, the Genomics Education Partnership (thegep.org) developed a curriculum involving web-based tools that allow undergraduates to engage in authentic course-based research focused on manually annotating genes in non-model species (Rele et al., 2023). The orthologous gene models, including the one presented here, then provide a reliable basis for further evolutionary genomic analyses when made available to the scientific community. The gene ortholog described here in *D. funebris* for *ATP binding cassette subfamily B member 7* ( *ABCB7* ), a member of the ABC-transporter gene family, was characterized as part of a developing dataset for a comparative study of detoxification gene families in the *immigrans* - *tripunctata* radiation of the genus *Drosophila* .” (Williams et al., 2026) “ *Drosophila funebris* (Fabricius, 1787) is a member of the *funebris* species group, which occurs in the *immigrans-tripunctata* radiation of the *Drosophila* subgenus (Bächli, 2005; ICZN, 2010). It is also the type species of the genus *Drosophila* . This species is a globally distributed human commensal (Grimaldi, 2022). While *D. funebris* feeds on shelf fungi and fruit (Kimura et al., 1977; Prigent et al., 2003), it does not tolerate the mushroom toxin α-amanitin (Stump et al., 2011; Erlenbach et al., 2023).” (McDonald et al., 2026) ATP-binding cassette (ABC) transporters constitute one of the largest protein superfamilies across all domains of life, functioning as primary-active transporters that use the energy of ATP hydrolysis to move a broad range of substrates across lipid membranes (Higgins, 1992; Dean et al., 2001). In the context of xenobiotic metabolism, ABC transporters mediate phase III detoxification by excreting toxins along with phase I and phase II metabolites out of cells. Their overexpression is a recurrent mechanism of insecticide and multidrug resistance in insects (Merzendorfer, 2014; Dermauw and Van Leeuwen, 2014). *ATP-binding cassette subfamily B member 7* ( *ABCB7* ) is an ABC transporter gene in the mitochondrial ATM subfamily and a component of the iron–sulfur (Fe–S) cluster assembly pathway. It is predicted to export a sulfur-containing precursor required for cytosolic Fe–S cluster maturation (Dermauw and Van Leeuwen, 2014). The *ABCB7* transcript is maternally deposited and broadly expressed through embryogenesis (Tomancak et al., 2002). Ubiquitous RNAi knockdown is lethal, consistent with an essential role in development (Öztürk-Çolak et al., 2024).

We propose a gene model for the *D. funebris* ortholog of the *D. melanogaster* *ATP binding cassette subfamily B member 7* ( *ABCB7* ) gene. The genomic region of the ortholog corresponds to the GeMoMa FBtr0072755_R0 gene prediction in the ASM1890182v1 Genome Assembly of *D. funebris* ( GCA_018901825.1 – Kim et al., 2021). This model is based on mixed sex, adult RNA-Seq data from *D. funebris* (Erlenbach et al., 2023; https://doi.org/10.5061/dryad.hdr7sqvq2) and *ABCB7* in *D. melanogaster* using FlyBase release FB2024_02 ( GCA_000001215.4 ; Öztürk-Çolak et al., 2024).****

***Synteny***

The reference gene, *ABCB7 ,* occurs on chromosome 3L in *D. melanogaster* and nested within it is *CG33230 . ABCB7* is flanked upstream by *CG13926* and *Oxoglutarate dehydrogenase 2* ( *Ogdh2* ). Downstream, *ABCB7* is flanked by *gamma-glutamyl carboxylase* ( *GC* ), *CG13928* which is nested in *GC* , and *neuronal Synaptobrevin* ( *nSyb* ). The *tblastn* search of *D. melanogaster* ABCB7-PA (query) against the *D. funebris* genome assembly (GenBank Accession: GCA_018901825.1 ; database) placed the putative ortholog of *ABCB7* within contig_110 ( JAEIFK010000108 .1) which corresponds to the GeMoMa gene prediction FBtr0072755_R0 (E-value: 0.0; identity: 87.12% as determined by *blastp* ). Nested within the putative ortholog is the FBtr0072757 GeMoMa prediction that corresponds to *CG33230* (E-value: 0.0; percent identity: 67.25% as determined by *blastp* ). The putative ortholog is flanked upstream by the GeMoMa gene predictions FBtr0072823_R0, FBtr0091793_R0, and FBtr0072824_R0 (nested in FBtr0091793_R0), which correspond to *CG13926* , *CG33791* , and *CG12105* in *D. melanogaster* (E-value: 5e-58, 0.0 and 0.0; identity: 87.37%, 67.92%, and 47.44%, respectively, as determined by *blastp* ; Figure 1A ; Altschul et al., 1990). The putative ortholog of *ABCB7* is flanked downstream by the GeMoMa predictions FBtr007281_R0 and FBtr0072822_R0, which correspond to *GC* and *CG13928* in *D. melanogaster* (E-value: 0.0 and 6e-141; identity: 70.1% and 80%, respectively, as determined by *blastp* ). The putative ortholog assignment for *ABCB7* in *D. funebris* is supported by the following evidence: The gene predictions surrounding the *ABCB7* ortholog are mostly orthologous to the genes at the same locus in *D. melanogaster* , gene expression data corresponds with each prediction, and local synteny is largely conserved, supported by E-values and percent identities. As such, we conclude that the GeMoMa prediction FBtr0072755_R0 represents an ortholog of *ABCB7* in *D. funebris* ( Figure 1A ).

***Protein Model***

*ABCB7* in *D. funebris* has seven coding sequences (CDS) within its genomic sequence. The first unique protein sequence (ABCB7-PA) is translated from one mRNA isoform (ABCB7-RA; Figure 1B ). The other unique protein sequence (ABCB7-PB) is also translated from a single mRNA isoform (ABCB7-RB; Figure 1B ). Relative to the ortholog in *D. melanogaster* , the CDS number and protein isoform count are conserved. The sequence of ABCB7-PA in *D. funebris* has 86.2% identity (92.7% similarity) with the protein-coding isoform ABCB7-PA in *D. melanogaster* , as determined by *blastp* ( Figure 1C ). Additionally, the sequence of ABCB7-PB in *D. funebris* has 84.4% identity (91.1% similarity). This level of divergence is not surprising given that *D. funebris* and *D. melanogaster* belong to two separate subgenera ( *Drosophila* and *Sophophora* respectively) that diverged approximately 45-60 MYA (Russo et al., 1995; Tamura et al., 2004; Obbard et al., 2012). Coordinates of this curated gene model are archived in the CaltechDATA repository (see “Extended Data” section below).

## Methods

The annotation methods used in this project are adapted from those described in Rele et al. (2023), which includes algorithms, database versions, and citations for the complete annotation process developed for the Pathways Project. The methods for the current project are detailed in brief below with notes on significant differences between this protocol and the one described in Rele et al. (2023). The students use the GEP instance of the UCSC Genome Browser v.435 (https://gander.wustl.edu; Kent et al., 2002; Raney et al., 2024) to examine the genomic neighborhood of their reference detoxification gene in the *D. melanogaster* genome assembly (Aug. 2014; BDGP Release 6 + ISO1 MT/dm6). Students obtain the protein sequence for the *D. melanogaster* target gene for a given isoform and use a *tblastn* search of the sequence against their target *Drosophila* species genome assembly ( *D. funebris* ( GCA_018901825.1 – Kim et al., 2021)) on the NCBI BLAST server (https://blast.ncbi.nlm.nih.gov/Blast.cgi, Altschul et al., 1990) to identify the putative ortholog location. Students compare the genomic neighborhood of the putative ortholog to that of the reference gene in *D. melanogaster* . This local synteny analysis includes a minimum of two upstream and two downstream genes relative to the potential ortholog. As no RefSeq protein data is available for these species, comparisons are based on gene predictions that correlate with gene expression data in the putative ortholog neighborhood. Using the multiple alignment tracks feature in the Genome Browser, students examine other sets of genomic evidence, including Spaln alignment of *D. melanogaster* proteins, multiple gene prediction tracks (e.g., GeMoMa, Augustus, NSCAN PASA-EST), and mixed sex RNA-Seq adult expression data from the target species generated by Erlenbach et al. (2023; https://doi.org/10.5061/dryad.hdr7sqvq2). Information on the genomic structure information (e.g., CDSs, intron-exon number, number of isoforms) for the reference gene in *D. melanogaster* is retrieved using Gene Record Finder (https://gander.wustl.edu/~wilson/dmelgenerecord/index.html; Rele et al *.,* 2023). To determine approximate splice sites within the target gene, a *tblastn* search using the CDSs from the *D. melanogaste* r reference gene against the putative ortholog location (10kb up- and downstream of the target gene prediction). Coordinates of the CDS(s) are refined by examining aligned RNA-Seq data, identifying canonical splice site sequences, and ensuring the maintenance of an open reading frame. Students confirm the biological validity of their target gene model using the FlySeq Gene Model Checker (https://gander2.wustl.edu/~wilson/genechecker-flyseq/), which compares the hypothesized target gene model's structure and translated sequence against the *D. melanogaster* reference gene. At least two independent models for this gene are generated. These models are reconciled by a third independent researcher to produce the final model presented here. Note: comparison of 5' and 3' UTR sequence information is not included in this GEP CURE protocol.

## Data Availability

Description: Zipped archive containing FASTA, PEP, and GFF of ABCB7 model in D. funebris. Resource Type: Model. DOI: https://doi.org/10.22002/pr8ks-0fg53
